# Development, Characterization and In Vivo Pharmacokinetic Assessment of Rectal Suppositories Containing Combination Antiretroviral Drugs for HIV Prevention

**DOI:** 10.3390/pharmaceutics13081110

**Published:** 2021-07-21

**Authors:** Kunal Jhunjhunwala, Charles W. Dobard, Sunita Sharma, Natalia Makarova, Angela Holder, Chuong Dinh, James Mitchell, Lin Wang, Junmei Zhang, Sravan Kumar Patel, Walid Heneine, Lisa C. Rohan

**Affiliations:** 1 Department of Pharmaceutical Sciences, School of Pharmacy, University of Pittsburgh, Pittsburgh, PA 15213, USA; ksj13@pitt.edu (K.J.); lwang@mwri.magee.edu (L.W.); junmei.zhang@pitt.edu (J.Z.); patels10@mwri.magee.edu (S.K.P.); 2 Magee-Womens Research Institute, Pittsburgh, PA 15213, USA; 3 Laboratory Branch, Division of HIV/AIDS Prevention, Centers for Disease Control and Prevention, Atlanta, GA 30333, USA; cdobard@cdc.gov (C.W.D.); fys6@cdc.gov (S.S.); wcn5@cdc.gov (N.M.); fka5@cdc.gov (A.H.); vul9@cdc.gov (C.D.); zjl9@cdc.gov (J.M.); wmh2@cdc.gov (W.H.)

**Keywords:** tenofovir alafenamide fumarate (TAF), tenofovir diphosphate (TFV-DP), elvitegravir (EVG), prodrug, rectal suppository, non-human primate (NHP), HIV prevention, pre-exposure prophylaxis (PrEP), rectal microbicide (RM), pharmacokinetics (PK)

## Abstract

Receptive anal intercourse (RAI) contributes significantly to HIV acquisition underscoring the need to develop HIV prevention options for populations engaging in RAI practices. We explored the feasibility of formulating rectal suppositories with potent antiviral drugs for on-demand use. A fixed-dose combination of tenofovir (TFV) and elvitegravir (EVG) (40 mg each) was co-formulated in six different suppository bases (three fat- and three water-soluble). Fat-soluble witepsol H15 and water-soluble polyethylene glycol (PEG) based suppositories demonstrated favorable in vitro release and were advanced to assess in vivo pharmacokinetics following rectal administration in macaques. In vivo drug release profiles were similar for both suppository bases. Median concentrations of TFV and EVG detected in rectal fluids at 2 h were 1- and 2-logs higher than the in vitro IC50, respectively; TFV-diphosphate levels in rectal tissues met or exceeded those associated with high efficacy against rectal simian HIV (SHIV) exposure in macaques. Leveraging on these findings, a PEG-based suppository with a lower dose combination of tenofovir alafenamide (TAF) and EVG (8 mg each) was developed and found to achieve similar rectal drug exposures in macaques. This study establishes the utility of rectal suppositories as a promising on-demand strategy for HIV PrEP and supports their clinical development.

## 1. Introduction

According to a recent report by the Centers for Disease Control and Prevention (CDC), 37,881 new HIV infections were diagnosed in the United States in 2018 [1]. Of these infections, the majority (~26,000, 69%) were among men who have sex with men (MSM). The higher risk for HIV infection associated with receptive anal intercourse (RAI) compared to vaginal intercourse may be explained by anatomical and physiological differences between the rectum and vagina. For instance, the rectal cavity is lined with a single layer of columnar cells compared to multiple layered stratified squamous epithelium of the vagina, and the thickness of the rectal epithelium is only about one-tenth of the vaginal epithelium (~25 and 215 µm, respectively) [2]. Unlike the vagina, the rectum is an open-ended tract with a large surface area [2,3], making the rectal compartment more susceptible to damage and thus increasing the probability of viral entry [4]. Furthermore, the rectal columnar epithelium is heavily populated with HIV-1 target cells compared to the multilayered vaginal epithelium, which does not possess a large presence of target cells at the surface [2,3]. Therefore, the rectal compartment requires a different product development strategy compared to the vagina [5,6]. Growing evidence in the literature indicates a compelling need to develop safe and effective rectal microbicides (RM) [7,8,9,10]. Topical administration of products such as gels, douches, fast-dissolving suppositories or inserts containing potent antiretroviral drugs (ARVs) is a promising strategy for on-demand pre- or post-exposure prophylaxis.

The first-generation RMs, which were gel formulations containing one or two active pharmaceutical ingredients (APIs), provided important data on using this strategy to prevent rectal HIV transmission. Cranage et al. found that 1% tenofovir (TFV) gel administered rectally to macaques up to 2 h prior to a single high-dose rectal simian immunodeficiency virus (SIV) challenge protected six of nine animals (67% protective efficacy) [11]. Dobard et al. evaluated a rectal-specific gel formulation containing 1% TFV, 1% Maraviroc (MVC), or a TFV (1%)/MVC (1%) combination and found that all three gel formulations provided 82% efficacy in a repeat rectal challenge macaque model [12]. These data provided proof-of-concept efficacy for RMs, supporting the development of improved dosage forms that have high biological efficacy and user desirability. Here, we report on the development of rectal suppositories with a two-drug combination. Suppositories offer greater flexibility in terms of shape and size, which facilitate their insertion and retention in the cavity and have higher drug loading capacity compared to gels. They are designed to be discreet, dissolve quickly, and administered without an applicator. A variety of suppository bases can be used to accommodate a wide range of APIs including those with high hydrophilicities or lipophilicities [13]. Additionally, melting point properties can be easily adjusted if necessary and ingredients such as surfactants can be added to modify drug release rates [14].

In this work, a panel of rectal suppositories containing both fat- and water-soluble bases along with antiretroviral (ARV) agents were manufactured, and their physicochemical attributes were thoroughly investigated. A detailed pharmacokinetic evaluation was also conducted in macaques following rectal dosing. A combination of two ARVs from different classes was selected to afford a wide dosing window for pre- or post-coital applications for more flexible dosing options for users. The first drug, TFV or its prodrug tenofovir alafenamide (TAF) (evaluated at a 5× lower dose considering the significantly higher antiviral potency of TAF compared to TFV [15,16,17,18,19,20,21,22,23,24,25,26,27,28]) blocks reverse transcription, which is an early step in the HIV replication cycle. The second drug elvitegravir (EVG) is an integrase strand-transfer inhibitor that prevents integration of HIV proviral DNA into host DNA, a step that occurs after reverse transcription and at least 6 h after virus entry into a host cell [29]. Inserts containing TAF and EVG were developed by CONRAD for on-demand vaginal or rectal use for HIV pre- and post-exposure prophylaxis [28,30,31]. In preclinical studies using normal cycling pigtail macaques, vaginal Simian-Human Immunodeficiency Virus (SHIV) exposure 4 h before or after vaginal administration of inserts co-formulated with TAF (20 mg) and EVG (16 mg) resulted in an estimated efficacy of 92% and 100%, respectively [28,30], supporting the on-demand utility of the drug combination for PrEP. Additional studies in macaques showed that rectal application of the same TAF/EVG inserts resulted in high tissue EVG and TFV-DP levels at 4 h that were within range of those associated with vaginal protection [31]. Phase 1 clinical trials evaluating the safety, pharmacokinetics, and pharmacodynamics of vaginal and rectal administration of TAF/EVG inserts are currently underway (NCT03762772 and NCT04047420 (MTN-039), respectively). In this work, we developed suppositories co-formulated with TFV (40 mg)/EVG (40 mg) or TAF (8 mg)/EVG (8 mg) as an alternative rectal dosage form with a long history of use and familiarity. The in vitro and in vivo assessments in a non-human primate model led to the identification of optimal TFV/EVG and TAF/EVG suppository formulations that can be advanced for clinical development.

## 2. Materials and Methods

Tenofovir (TFV) used in the formulations was purchased from Wuxi, AppTec (Shanghai, China). Tenofovir alafenamide fumarate (TAF) was provided by Laurus Labs to the Centers for Disease Control and Prevention (CDC) (Atlanta, GA, USA). Elvitegravir (EVG) was provided by Gilead Sciences, Foster City, CA (for the TFV + EVG suppositories) or by Hetero Drugs (Hyderabad, India) to CDC (for the TAF + EVG suppositories). The fat-soluble base witepsol was a gift from IOI Oleo GmbH (Hamburg, Germany), and suppocire base was from Gattefosse (Paramus, NJ, USA). Cocoa butter and three Polyethylene-Glycol (PEG) polymers of different molecular weights of 3350, 1000, 400 were purchased from Spectrum Chemical, Gardena, CA, USA. The plastic disposable suppository molds were purchased from Professional Compounding Centers of America (PCCA), Houston, TX, USA. Tetra-butyl ammonium bromide, potassium mono- and di-phosphate salts used in the HPLC assays were purchased from Fisher Scientific, NJ, or J.T. Baker, NJ.

### 2.1. Manufacture of Rectal Suppositories

Suppositories were manufactured by fusion molding technique using disposable plastic suppository molds. TFV and EVG loaded suppositories were manufactured with three fat-soluble and three water-soluble bases. Fat-soluble suppository bases were melted in a container placed in a heated water bath (~45 °C) and drugs were added to the melt in small portions to avoid the formation of aggregates. The base-drug mixture was subjected to slow handshaking or an overhead mixer depending on the batch size. Once uniformity was achieved, the suspension of the drugs in the suppository base was slowly poured into disposable plastic suppository molds (1–1.5 g). PEG suppositories were manufactured by mixing PEG polymers of different molecular weights. They were weighed and melted in increasing order of melting points such that the temperature of the molten mixture was maintained between 65–70 °C after the addition of each polymer. Drugs were added in small proportions to avoid aggregate formation. This was followed by pouring the drug-loaded molten mass into disposable plastic suppository molds up to the shoulder. The suppositories were allowed to cool naturally to room temperature until they were completely solidified. TAF and EVG suppositories were also manufactured using the same procedure, but with a PEG base only.

### 2.2. Suppository Characterization

Visual inspection was conducted to examine the color and surface texture of the suppositories. Each unit was visually inspected for cracks and pits that can be caused by air entrapment.

Weight variation of the suppositories was determined by weighing individual units using an analytical balance. The deviation of each unit from the average of the batch was calculated and the acceptance criteria were set up as 5% relative standard deviation (RSD).

Hardness was measured using Texture Analyzer TA.XT.plus (Stable Micro Systems, Surrey, UK), which was operated using the “Exponent” software. The conical side of the suppositories was trimmed to obtain flat surfaces on both sides to facilitate measurement. The suppositories were placed on a flat platform (TA-90) and tested using a flat surface probe (TA-58). The height between suppository and probe was calibrated to 10 mm, and the length traveled by the probe after contacting the suppository surface was set to 5 mm to ensure the suppository was broken completely. The force required to break the suppository (highest peak on the graph) was considered as the breaking point to represent the hardness. It should be noted that due to their soft nature, the PEG suppositories lost their shape when compressed by the texture analyzer probe instead of breaking into two halves. Thus, the maximum force required at 5 mm to compress the suppository was recorded as the hardness for the PEG suppositories.

Thermal transition temperatures of suppositories were measured by a Differential Scanning Calorimeter (DSC), Mettler Toledo (Columbus, OH, USA). Approximately 2 to 8 mg of suppository sample was weighed in an aluminum crucible and sealed using a sealer. The nitrogen flow rate was set at 50 mL/min. The temperature ranges were 25–50 °C and 25–60 °C for the fat- and PEG-based suppositories, respectively. The scan rate was 5 and 1 °C/min for the fat- and PEG-based suppositories, respectively. The heat flowing through this crucible was compared to an empty crucible (as a reference) and the change in heat flow was measured. The endothermic peak was recorded as the thermal transition temperature of the sample.

Disintegration time (DT) for suppositories was determined using Electrolab Disintegration tester apparatus (Model no: ED 2L, Version 1.1, Betatek, ON, Canada). The medium was 700 mL of 1X PBS at pH 7.4 heated to 37 ± 0.5 °C. The time point at which the PEG-based suppositories completely dissolve and the fat-soluble-based suppositories melt was recorded as the DT.

In vitro release (IVR) of suppositories was conducted in a Distek T2100 series dissolution apparatus (Distek Inc., North Brunswick, NJ, USA). USP Apparatus Type 1 basket was utilized for this experiment. The distance between the inner surface of the vessel and the basket was manually adjusted to 25 ± 2 mm. Sink conditions were maintained, and the dissolution media were 500 mL 5% SDS and 500 mL 1% SDS for the TFV + EVG and TAF + EVG suppositories, respectively. Dissolution was conducted at 37 °C and 100 RPM agitation. Two mL aliquots were collected at 15, 30, 45, 60, 75, 90, 105, 120, 150 and 180 min and replenished with fresh medium. These samples were filtered using a syringe and 0.22 µm PTFE syringe filters, diluted and analyzed using HPLC.

Drug content of TFV, TAF and EVG was analyzed from the suppositories using different analytical methods as described below.

TFV: A Dionex Ultimate 3000 High-Performance Liquid Chromatography (HPLC, Thermofisher, Sunnyvale, CA, USA) system with UV or PDA detector was used to quantify TFV from suppositories and assess the drug content uniformity at 260 nm. Separations were achieved on a Phenomenex Gemini C18 (150 × 4.6 mm) column fitted with a Phenomenex C18 guard column at 25 °C. The mobile phase was a mixture of 80% 10 mM K_2_HPO_4_ + 2 mM *t*-butylammonium bisulfate (tBAHS) adjusted to pH 5.7 using 30% phosphoric acid and 20% methanol. The retention time for TFV was 5.3 ± 0.2 min at 1 mL/min. A calibration curve was prepared in the range of 5–200 µg/mL. Each suppository was placed in a 20 mL volumetric flask and melted at 65 °C for PEG-based and 45 °C for fat-based suppositories using a water bath. The volume was made up to 20 mL with 10% methanol and the drug was extracted by vigorously vortexing the samples. The mixture was allowed to cool down to room temperature so that the fat-soluble bases became separated from the solvent. Then, 1 mL aliquots were withdrawn and centrifuged at 10,000 rpm for 10 min. The supernatant was filtered using 0.22 µm PTFE syringe filters. Suitable dilutions were made with 10% methanol and analyzed using HPLC.

TAF: An Ultimate 3000 (Dionex) HPLC system with UV or PDA detector was used for TAF analysis. Analysis was conducted on a Phenomenex Gemini C18 150 × 4.6 mm column along with a guard column (Phenomenex C18) at 25 °C. Then, 25 mM potassium phosphate + 5 mM tetra-ammonium butyl bromide (at pH 6): methanol (80:20) (A) and acetonitrile (B) were used as the mobile phase using a gradient method. The gradient elution started with 5% B, increased to 25% B from 6 to 8 min with no change up to 12 min, returned to 5% B at 14 min with linear change, and then remained at 5% B between 14 and 17 min. The flow rate was 1 mL/min. TAF was eluted at 14 ± 0.2 min. A calibration curve was made with a concentration range between 10–200 µg/mL. The PEG-based TAF suppositories were melted in a 20 mL volumetric flask at 65 °C using a water bath and dissolved in 100% methanol. These samples were diluted within a suitable range using 25% acetonitrile, centrifuged and filtered using 0.22 µm PTFE syringe filters. The final dilutions were made using methanol, acetonitrile and water at the ratio 10:22:68 and the filtered solutions were analyzed using HPLC.

EVG: The HPLC system used to develop and validate the assay for EVG was an Ultimate 3000 (Dionex) equipped with a photodiode array detector. Separations were achieved on a Synergi 4 μ Polar column (150 × 2.00 mm, RP 80 Å; Phenomenex) fitted with a guard column (Phenomenex Gemini C18) at 37 °C with a flow rate of 0.5 mL/min. The mobile phase was composed of 0.2% formic acid (FA) in water and 0.2% FA in acetonitrile. The retention time of EVG was 7.1 ± 0.2 min. A calibration curve was made within the concentration range of 1–100 µg/mL. Each suppository was placed in a 20 mL volumetric flask and melted at 65 °C for PEG-based and 45 °C for fat-soluble-based suppositories using a water bath. The volume was made up to 20 mL with acetonitrile and the drug was extracted by vigorously vortexing the samples. The solution was allowed to cool down to room temperature so that the fat-soluble bases became separated from acetonitrile. Then, 1 mL aliquots were withdrawn and centrifuged at 10,000 rpm for 10 min. The supernatant was filtered using 0.22 µm PTFE syringe filters prior to HPLC analysis. When dilutions were necessary, samples were diluted using 0.2% FA in water and 0.2% FA in acetonitrile at a 70:30 ratio.

### 2.3. Humane Care Guidelines

All animal protocols were approved by the Centers for Disease Control and Prevention (CDC) Institutional Animal Care and Use Committee (IACUC, #2708, 30 November 2015). Rhesus macaques (Macaca mulatta) were cared for by CDC veterinarians in compliance with the Guide for the Care and Use of Laboratory Animals 8th Ed [32].

### 2.4. In Vivo PK Following Rectal Suppository Dosing

The pharmacokinetic profile of rectal suppositories containing a fixed-dose combination of TFV and EVG (40 mg each) was evaluated in a cross-over study design using 6 rhesus macaques. Briefly, two groups of 3 macaques received TFV/EVG (40/40 mg) suppositories formulated with witepsol H15 (Grp1; *n* = 3) or PEG (Grp2; *n* = 3) base. At baseline, suppositories were administered digitally approximately 1–2 cm into the rectum and drug levels were measured at 2, 5 or 24 h post-dosing. To minimize animal discomfort following biopsy collections, rectal suppositories were administered once weekly over 6 weeks to measure drug levels at each time point (up to 6 measurements per base). To account for inter- and intra-variability between animals, study groups were switched weekly and macaques received suppositories with a different base. Animals received rectal washes with sterile saline (3–4 mL) up to 1 h prior to rectal dosing to limit interference in drug absorption due to fecal contamination.

The PK profile of PEG-based rectal suppositories containing TAF and EVG (8 mg each) was evaluated in 6 rhesus macaques using a similar sample collection schedule (2, 5, and 24 h) as described above with slight modifications. To increase the sample size of drug measurements, rectal dosing and sample collections for the 2- and 5-h time points were repeated in the same animals following a one-week washout period between dosing. Drug levels at 24 h were measured on a separate occasion following rectal dosing of the same animals. Drug distribution of TFV-DP in colorectal tissues was investigated in another group of SHIV-infected macaques that were sacrificed 2 h (*n* = 4) and 24 h (*n* = 4) after receiving a single TAF/EVG (8/8 mg) suppository. Intact colorectal tissue (~30 cm) excised at the time of necropsy was gently rinsed with saline to remove feces and dissected into 5 cm tissue sections from the anal margin to the colon (0–5, 6–10, 11–15, and 16–20 cm). Rectal lymphocytes were isolated from each tissue section by established enzymatic extraction methods previously described [12].

### 2.5. Specimen Collections

Whole blood was collected into Cell Preparation Tube (CPT) tubes for the isolation of plasma. Rectal fluids were collected by inserting a pre-weighed Weck-Cel spear rectally (3–5 cm) and maintaining it in place for 1–2 min to allow for absorption. The spear was transferred to a collection tube, weighed, and immediately snap-frozen on dry ice and stored at −80 °C. Rectal biopsies were collected 2–4 cm from the anal margin using 3.7-mm biopsy forceps (Radial JawTM 3 Biopsy Forceps, Boston Scientific, Marlborough, MA, USA) and transferred to tared screwcap tubes and the total weight was recorded (median 11.5 mg). The tubes were snap-frozen on dry ice and stored at −80 °C.

### 2.6. Analysis of Drug Concentrations in Plasma, Rectal Fluids, and Rectal Biopsies Biological Matrices

The concentrations of EVG, TAF, and TFV were measured by HPLC–MS/MS as previously described [33]. Briefly, analytes were extracted from 0.1 mL of plasma, a single Weck-Cel spear tip, or 0.25 mL of tissue homogenate by protein precipitation using 500 µL of methanol containing an internal standard (^13^C-labeled TFV, deuterium-labeled TAF (Moravek, Brea, CA, USA), deuterium-labeled EVG (EVG-d6) (Toronto Research Chemicals, Toronto, ON, Canada)). Drug concentrations were estimated from a standard curve with a range of 0.5–2000 ng/mL using Analyst software. Drug concentrations measured in rectal spears were converted to ng/mL based on the net weight (assuming 1 µL = 1 mg). The lower limit of quantification (LLOQ) in plasma was 10 ng/mL for TFV and TAF and 5 ng/mL for EVG. The LLOQ in rectal fluids and tissue was 10 ng/mL and 1 ng/mg for each analyte, respectively. All calibration curves had R^2^ values greater than 0.99.

### 2.7. Analysis of Tenofovir Di-Phosphate (TFV-DP) Concentrations in Rectal Biopsies and Rectal Lymphocytes

Intracellular concentrations of TFV-DP in rectal tissue biopsies and rectal lymphocytes were measured by LC-MS-MS as previously described [12,34]. Briefly, minced rectal tissue biopsies and purified rectal lymphocytes (5 million cells) were lysed with 1 mL of cold 80% methanol containing 1000 ng of ^13^C-labeled TFV-DP as internal standard and stored at −80 °C until further analysis [12]. Calibration curves were generated from standards of TFV-DP spiked into 80% methanol over the range from 0.25 to 10 nM. The LLOQ for TFV-DP was 100 fmol/sample. All calibration curves had R^2^ values greater than 0.99.

### 2.8. Data and Statistical Analysis

The in vitro dissolution results are represented as mean ± standard deviation (SD), however, the in vivo PK data are represented as median ± range. GraphPad PRISM 7.0 was used to process and analyze the data. Statistical differences were calculated between suppository bases (PEG versus witepsol) as well as across time points using two-way ANOVA. *p* values ≤ 0.05 were considered statistically significant. These analyses were conducted for every biological matrix, i.e., rectal fluid, biopsies and plasma. Sidak’s multiple comparison post-hoc tests were conducted for in vivo PK data wherever required.

## 3. Results

We screened a series of suppository bases and ARV candidates to assess the feasibility of rectal suppositories for HIV prevention (unpublished data). Results from these initial studies led us to six suppository formulations (three water-soluble and three fat-soluble). Using this short-list of suppository bases, we co-formulated suppositories with 40 mg TFV and 40 mg EVG (TFV/EVG (40/40 mg)) as well as suppositories with 8 mg TAF and 8 mg EVG (TAF/EVG (8/8 mg)).

### 3.1. TFV/EVG (40/40 mg) Suppositories

#### 3.1.1. In Vitro Characterization

During the development phase, three fat-soluble bases of different grades and manufacturers, and three water-soluble bases containing PEG with different molecular weights were utilized to formulate six types of placebo suppositories. The suppository bases were selected using the decision tree provided by the manufacturers and compatibility with the physiochemical properties of the APIs. The suppositories were bullet-shaped with a typical length of approximately 1 inch. The physical appearance of placebo suppositories was noted and each formulation was characterized by weight variation, hardness, melting point, and disintegration time (Table 1).

Weight variations. The weight variations were within 2% for each type of suppository (Table 1).

Hardness. The maximum force required to break or compress suppositories was used to evaluate hardness for fat-soluble or water-soluble bases, respectively. Among the three fat-soluble suppositories, hardness was ranked in the following order: witepsol H15 > suppocire A > cocoa butter (Table 1). Cocoa butter, a naturally occurring fat-soluble base, has the lowest melting point and different chemical composition compared to witepsol H15 and suppocire A and as a result, tends to break easily. In contrast, suppocire A and witepsol H15 consist primarily of glycerides, which have a higher melting temperature and are specifically designed for rectal and vaginal administration and to withstand pressure during handling and transportation. Among the three PEG-based suppositories, the ranking order of suppository hardness was PEG 8000/400 > PEG 3350/1000 > PEG 3350/1000/400 (Table 1).

Disintegration time. Another parameter used to evaluate the strength of the suppository bases was the disintegration time or the time required for complete disintegration at body temperature. A phosphate buffer saline (pH 7.4) solution was used to mimic the pH of the rectal fluid. As expected, suppositories with fat-soluble bases disintegrated relatively quickly (Table 1). In contrast, the water-soluble PEG-based suppositories dissolved at a much slower rate (7–15 min). The disintegration times of PEG-based suppositories ranked in the following order: PEG 8000/400 > PEG 3350/1000 > PEG 3350/1000/400 (Table 1).

Thermal transition temperatures. Measured by differential scanning calorimetry (DSC), the thermal transition temperatures of the witepsol H15 and cocoa butter suppositories were found to be at or below body temperature (Table 1). Two of the three PEG-based formulations showed thermal transition temperatures higher than body temperature (Table 1).

The bases (PEG and fat-soluble) most suitable for facile manufacturing, and with optimum physical properties were selected for drug incorporation. The dose selection of 40 mg for each API was based on data generated from previous preclinical macaque studies with gel formulations containing TFV or integrase inhibitors as well as clinical trials evaluating TFV gels [11,12,29,35,36]. The characteristics of the drug-loaded suppositories were similar to that of the placebo suppositories (hardness not tested due to the limited drug availability). The in vitro release of drug-loaded suppositories was evaluated.

**Figure 1:**
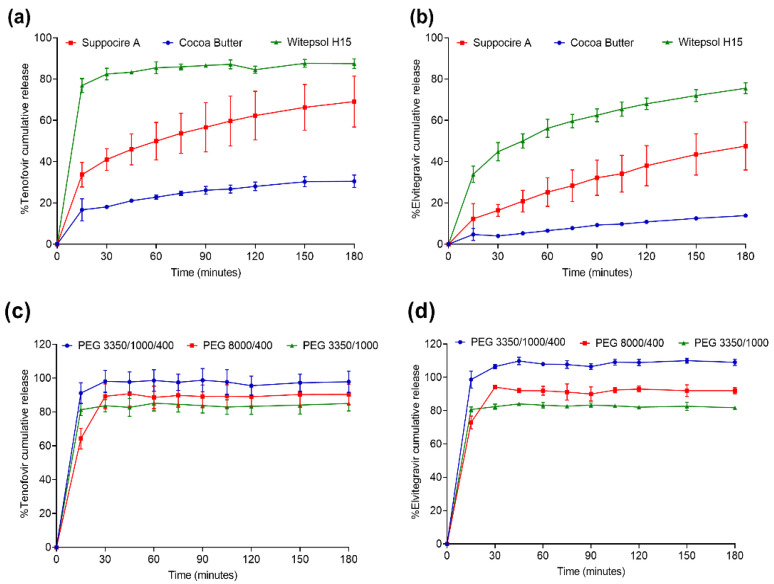
In vitro dissolution of tenofovir (**a**–**c**) and elvitegravir (**b**–**d**) from suppositories with three fat-soluble bases (**a**,**b**) and three PEG bases (**c**,**d**).

In vitro release. We next investigated if the physicochemical (hydrophilic/hydrophobic) properties of the two drugs influenced the dissolution rates from the suppositories. Due to the lipophilic nature of EVG, a surfactant (5% SDS) was added to the dissolution media to maintain sink conditions. All the suppository formulations were tested for the cumulative amount of drug released over a period of 3 h with pre-defined time intervals. TFV was consistently released at a faster rate compared to EVG in all the fat-soluble bases (Figure 1a,b). At 30 min, 82%, 41%, and 18% of TFV (*p* < 0.0001) was released from the witepsol H15, suppocire A and cocoa butter-based suppositories compared to only 44%, 16%, and 4% of EVG, respectively. EVG is highly lipophilic and thus is expected to have a stronger interaction with the fat-soluble suppository bases, which may explain the slower release rate.

The PEG-based suppositories were completely dissolved in the dissolution media within 15 min, indicating a rapid burst release (Figure 1c,d). Due to the rapid dissolution time, no significant differences (one-way ANOVA) were observed for the release of TFV (*p* = 0.5441) or EVG (*p* = 0.1723) from the different PEG-based suppositories. Notably, suppositories formulated with PEG 3350/1000/400 at a ratio of 60:30:10 resulted in the fastest drug release kinetics among the three PEG-based formulations.

#### 3.1.2. In Vivo Pharmacokinetic Assessment

The witepsol H15 based and PEG 3350/1000/400 based combination suppositories were chosen for the in vivo PK studies. Following rectal administration, blood, rectal swabs, and rectal biopsies were collected to measure EVG and TFV in plasma and rectal fluids. EVG, TFV and its pharmacologically active metabolite TFV-diphosphate (TFV-DP) were measured in tissue homogenates. The calculated C_max_, T_max_ and AUC_0–24_ parameters are summarized in Table 2.

**Figure 2:**
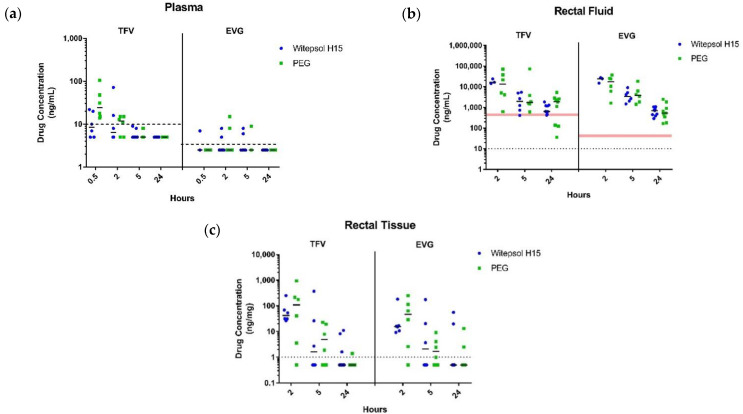
In vivo pharmacokinetic assessment following rectal administration of TFV/EVG (40 mg/40 mg) suppositories formulated in witepsol H15 or PEG base in macaques. TFV and EVG levels in plasma (**a**), rectal fluids (**b**), and rectal biopsies (**c**). Dotted line represents the lower limit of quantitation (LLOQ). Red shaded line represents in vitro IC_50_.

Plasma PK. Low and transient systemic exposures of TFV and EVG were observed following rectal dosing with witepsol- or PEG-based suppositories. Consistent with drug release kinetics observed in vitro, PEG-based suppositories resulted in faster release and higher concentrations of TFV in plasma compared to witepsol-based suppositories (Figure 2a). Median TFV levels following rectal dosing with PEG-based suppositories were highest at 30 min post-dosing (25 (14–106) ng/mL) and rapidly declined to undetectable levels by 5 h (Figure 2a). In contrast, median plasma TFV concentrations in animals dosed with witespol-based suppositories were all below the LLOQ (10 ng/mL). Similarly, median EVG concentrations were unquantifiable in plasma at all time points measured following rectal dosing with witepsol- or PEG-based suppositories (LLOQ = 5 ng/mL).

Rectal fluid PK. TFV concentrations in rectal fluids were similar 2 h after rectal dosing with TFV/EVG suppositories formulated in a witepsol- or PEG-base (16.3 and 13.4 µg/mL, respectively) and remained above the in vitro TFV IC_50_ (0.516 µg/mL) for up to 24 h post-dosing (0.64 and 1.86 µg/mL, respectively) (Figure 2b). The AUC_2–24h_ values for TFV were about 2-fold higher in animals dosed with PEG- compared to witepsol-based suppositories (26.4 and 12.2 µg × h/mL, respectively), suggesting an increase in TFV release and residence time with PEG-based suppositories. Unlike the findings in vitro, the EVG levels in rectal fluids were similar to TFV following rectal dosing with either witepsol- or PEG-based suppositories. Peak levels of EVG were also observed at 2 h (24.6 and 17.3 µg × h/mL, respectively) and remained about 1 log_10_ above the in vitro EVG IC_50_ (0.045 µg/mL) at 24 h post-dosing (0.69 and 0.53 µg/mL, respectively). Overall, the EVG levels in rectal fluids were similar in animals dosed with witepsol- and PEG-based suppositories (AUC_2–24h_ = 15.6 and 15.0 µg × h/mL, respectively).

Rectal tissue PK. The tissue concentrations of TFV and EVG over time following rectal dosing with witepsol or PEG-based TFV/EVG suppositories are shown in Figure 2c. Inter-animal variation in tissue drug concentrations at 2 and 5 h was considerable, particularly in animals treated with PEG-based suppositories. For each suppository base, TFV and EVG levels were similar and reached peaked concentrations within 2 h of rectal dosing (Figure 2c). The median rectal tissue concentrations at 2 h were consistently higher (~2–3-fold) for both TFV and EVG in animals dosed with PEG- compared to witepsol-based suppositories. The frequency of drug detection in rectal tissue was similar in both groups. Overall, 43% (9/21) and 48% (10/21) of samples were below the limit of quantification for TFV and EVG following rectal dosing with witepsol-based suppositories compared to 52% (11/21) with PEG-based suppositories, respectively.

**Figure 3:**
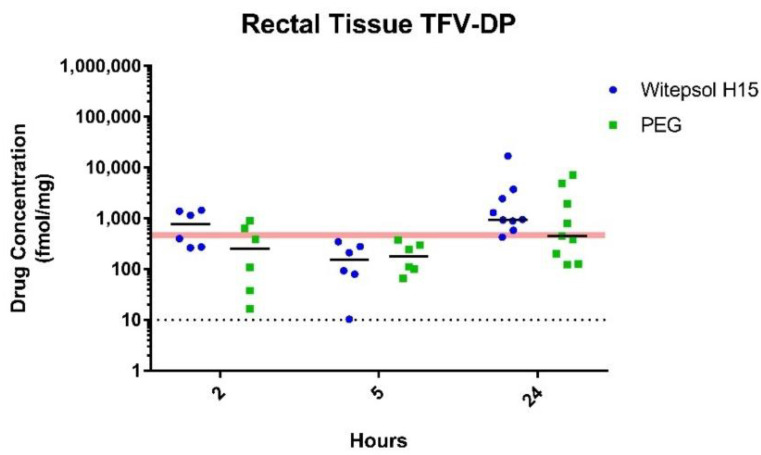
Intracellular TFV-DP levels in rectal biopsies following rectal administration of TFV/EVG (40 mg/40 mg) suppositories formulated in witepsol H15 or PEG base in macaques. Dotted line denotes the lower limit of quantitation (LLOQ). Red shaded line represents levels associated with in vivo protection (82% efficacy).

Intracellular TFV-DP. Intracellular levels of TFV-DP, the pharmacologically active form of TFV, were measured in tissue homogenates from the same rectal biopsies and time of collection described above. The median TFV-DP concentrations in rectal tissues following dosing with witepsol-based suppositories were high at 2 h (773 (265–1451) fmol/mg) and slightly increased over 24 h post-dosing (946.7 (425–16,999) fmol/mg) (Figure 3). Despite the higher TFV tissue concentrations in animals treated with PEG-based suppositories, median TFV-DP levels were about 2–3 times lower at 2 and 24 h (249 (16.5–888.3) and 454.5 (124.2–7128) fmol/mg, respectively) (Figure 3). Notably, the TFV-DP levels achieved 24 h following rectal dosing with PEG- and witepsol-based suppositories exceeded those associated with in vivo protection (82% efficacy; median TFV-DP = 415 (68–788) fmol/mg) in macaques dosed rectally with 1% TFV gel [12].

### 3.2. TAF/EVG (8 mg/8 mg) Suppositories

Based on the favorable in vitro and in vivo PK results with PEG-based suppositories containing TFV/EVG, the same PEG 3350/1000/400 base was used to formulate rectal suppositories containing a fixed-dose combination of TAF and EVG (8 mg each). As expected, the in vitro characterization profile of TAF/EVG (8 mg/8 mg) suppositories was similar to the TFV/EVG (40 mg/40 mg) suppositories including the appearance (white, opaque and sticky), weight variations (±5% RSD), and in vitro drug release. Notably, analysis of drug content revealed TAF/EVG suppositories contained slightly less than the target drug amounts (87.5% or 7 mg per drug).

#### In Vivo Pharmacokinetic Assessment

The PK profile of PEG-based TAF/EVG (8/8 mg) suppositories was determined using the same in vivo PK study design described for TFV/EVG suppositories. Following rectal administration of TAF/EVG suppositories, concentrations of EVG, TAF, and TFV were measured in plasma, rectal fluids, and rectal tissues and TFV-DP levels were measured in rectal biopsies. The calculated C_max_, T_max_ and AUC_2–24h_ parameters are summarized in Table 3.

Plasma PK. Analysis of drug exposures in plasma following rectal administration of TAF/EVG (8/8 mg) suppositories revealed that median levels of all three drug analytes (EVG, TAF, and TFV) were undetectable at all time points tested (data not shown). These results were not unexpected and consistent with lowering the dose by five-fold compared to the TFV/EVG (40/40 mg) suppositories.

**Figure 4:**
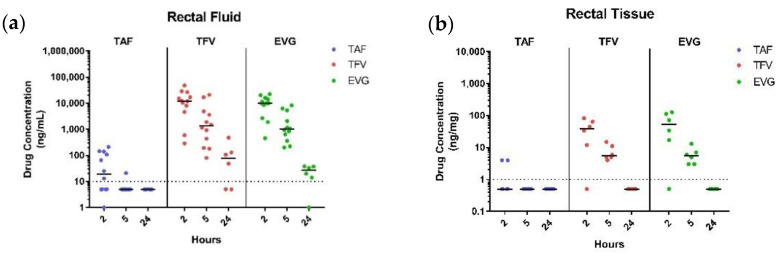
In vivo pharmacokinetics following rectal administration of PEG suppositories containing TAF/EVG (8 mg/8 mg). Drug concentration versus time plots in rectal fluid (**a**) and rectal biopsies (**b**). Dotted line denotes the lower limit of quantitation (LLOQ).

Rectal Fluid PK. EVG and TFV were consistently detected in rectal fluids up to 24 h post-dosing (Figure 4a). In contrast, median (range) TAF levels of TAF were low at 2 h (median = 19 (BLOQ−210) ng/mL) and 11/12 (92%) samples with levels below the limit of quantification by 5 h. However, TFV concentrations above IC_50_ were observed as early as 2 h and remained until 24 h, suggesting rapid in vivo conversion of TAF to TFV. The C_max_ values for TFV and EVG were similar (1.2 and 1.0 × 10^4^ ng/mL, respectively) and achieved within 2 h of suppository administration (Table 3), indicating rapid drug dissolution from suppositories.

Rectal tissue PK. TAF was mostly undetectable in rectal tissues for all time points, with 16/18 (89%) samples below the limit of quantification (Figure 4b). Consistent with rapid hydrolysis of TAF to TFV, TFV was frequently detected in rectal biopsies up to 5 h post-dosing. Moreover, the median C_max_ values for TFV tissue concentrations were similar to those for EVG at 2 h (39.5 and 53 ng/mg), suggesting a similar drug release rate from PEG-based TAF/EVG suppositories (Table 3). Median TFV and EVG tissue levels decreased seven to nine-fold by 5 h (5.5 and 5.6 ng/mg, respectively) and were below the limit of quantification in all animals 24 h post-dosing (Figure 4b).

**Figure 5:**
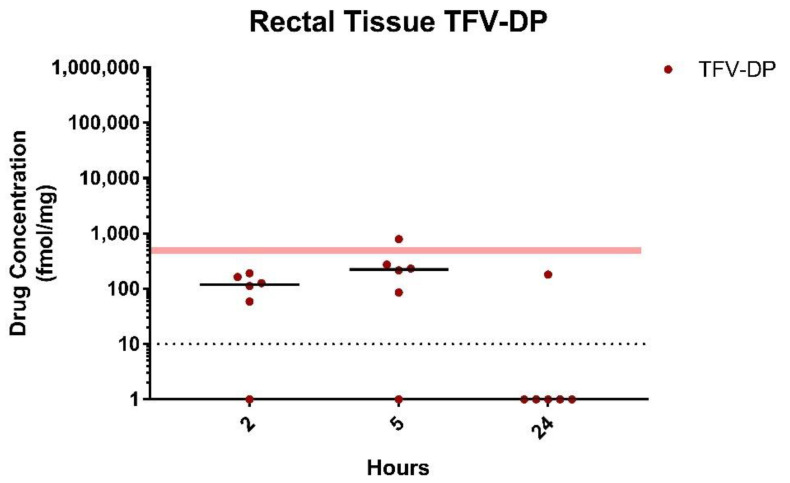
Intracellular TFV-DP levels following rectal administration of PEG suppositories containing TAF/EVG (8/8 mg). Dotted line denotes the lower limit of quantitation (LLOQ). Red shaded line represents levels associated with in vivo protection (82% efficacy).

**Figure 6:**
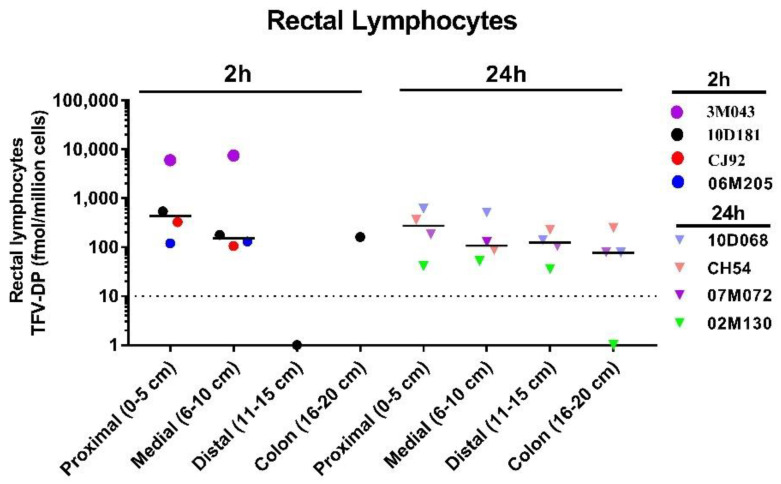
Intracellular TFV-DP levels in rectal lymphocytes were collected 2 and 24 h post-dosing with PEG suppositories containing TAF/EVG (8/8 mg). Dotted line denotes the lower limit of quantitation (LLOQ). Each color/symbol represents an individual animal.

Intracellular TFV-DP. Median TFV-DP levels in rectal tissue homogenates following rectal dosing with TAF/EVG (8/8 mg) suppositories were 191 and 224 fmol/mg at 2 and 5 h, respectively (Figure 5). Importantly, these levels were similar to those seen at 2 and 5 h following rectal dosing with PEG-based suppositories containing 40 mg of TFV (249 and 178 fmol/mg, respectively). Notably, except for one animal, TFV-DP levels were below the limit of quantification in rectal biopsies collected 24 h post-dosing. The low TFV-DP tissue levels likely reflect the low TAF and TFV exposures detected in rectal tissues 24 h post-dosing. Terminal studies were performed in SHIV-infected macaques to better assess drug distribution and TFV-DP levels in lymphocytes isolated from rectal tissues collected proximal and distal to the drug dosing site. Macaques were treated rectally with TAF/EVG (8/8 mg) suppositories and rectal tissues were collected at necropsy in animals sacrificed at 2 h (*n* = 4) or 4 h (*n* = 4) post-dosing. While not all tissue sections from each animal yielded enough cells for downstream intracellular drug analysis, TFV-DP was consistently detected in the lymphocytes extracted from tissue sections collected up to 20 cm from the anal margin. Within 2 h post-dosing, the median TFV-DP levels were 434.5 (120–6060), 155 (106–7517), BLOQ, and 163 fmol/10^6^ cells in lymphocytes collected from tissues sections of 0–5, 6–10, 11–15, and 16–20 cm, respectively (Figure 6). Notably, one animal had TFV-DP levels in rectal lymphocytes collected 0–5 and 6–10 cm (6060 and 7517 fmol/10^6^ cells, respectively) that were two to four times higher than those detected in lymphocytes collected at 2 h from the same site and shown to be associated with in vivo protection by rectal TFV gel (1389 and 3588 fmol/10^6^ cells, respectively) [12]. When comparing drug concentrations at 24 h post-dose, TFV-DP levels were lower but less variable for proximal to distal tissue sections (median = 274.4 (41.7–608), 108 (52.3–513), 123.5 (36–230), and 78.5 (BLOQ-251) fmol/10^6^ cells, respectively) (Figure 6). Taken together, these findings demonstrate rapid dosing and accumulation of TFV-DP in HIV target cells throughout the rectal tissue that extends well beyond the site of administration.

## 4. Discussion

We describe novel suppository formulations for delivering TFV or TAF in combination with EVG to rectal tissues for HIV prevention. The suppositories are designed for on-demand pre- or post-coital use by incorporating ARVs that block early and late steps in the HIV replication cycle. This design attempts to address limitations on timing seen in previous studies with rectal TFV gel, which demonstrated loss of post-exposure efficacy when macaques were dosed rectally as short as two hours after the SIV challenge [11]. Our PK assessments in macaques confirmed the rapid dissolution of suppositories and optimal tissue dosing that achieved previously established benchmarks for protection, thus supporting further preclinical and clinical advancement of the products.

We screened three water-soluble and three fat-soluble suppository bases to identify optimal bases for the suppository platform. Initial screening for physical, visual, and tactile attributes utilized placebo suppositories. Among the fat-soluble bases, witepsol H15 demonstrated the most favorable characteristics, namely the optimal combination of hardness and low melting temperature. Witepsol H15 also demonstrated rapid drug release of hydrophilic and lipophilic APIs (TFV and EVG, respectively) compared to suppocire A and cocoa butter. PEG polymers are readily water-soluble and ideal for suppository formulations, and as such displayed minimal differences. Despite challenges of differentiating the drug release kinetics of the PEG-based suppositories in vitro, PEG 3350/1000/400 (60:30:10) was selected as the best water-soluble candidate based on physical and tactile characteristics namely its smooth and visually pleasant texture.

Our in vivo PK studies demonstrated a favorable drug release profile including rapid dissolution and low systemic drug exposures following rectal dosing with witepsol- or PEG-based TFV/EVG suppositories. The concentrations of TFV and EVG in rectal fluids detected shortly after dosing (2 h post) were one to two orders of magnitude higher than the in vitro IC_50_ and irrespective of the formulation base, the median values remained above the IC_50_ for up to 24 h. Despite the high variability of TFV and EVG in rectal tissues, intracellular TFV-DP levels were consistently detected in rectal tissues, suggesting that other factors such as defecation may have contributed to the removal of drug from the rectal tissue. Importantly, the median TFV-DP levels in rectal tissues 24 h post rectal dosing with TFV/EVG (40/40 mg) suppositories were in range with those associated with in vivo rectal protection by rectal 1% TFV gel in a macaque model of rectal HIV transmission [12].

While the TFV/EVG (40/40 mg) suppositories were formulated to deliver the same TFV dose as the rectal TFV gel (40 mg), TFV exposures in rectal fluids following rectal dosing with witepsol- and PEG-based suppositories were orders of magnitude lower compared to those in macaques dosed rectally with TFV gel. However, despite the lower TFV exposure in rectal fluids, peak TFV concentrations in rectal tissues were similar to or exceeded those following dosing with rectal TFV gel. Similarly, the C_max_ TFV-DP levels achieved with PEG- or witepsol- based suppositories were also similar to or slightly higher than those following rectal dosing with 1% TFV gel, respectively [12]. These findings demonstrate that TFV can be effectively delivered rectally by suppositories and suggest suppositories formulated with TFV may provide high protection against rectal infection.

The PK profile of EVG was similar for both PEG- and witepsol- based suppositories and thus the high EVG levels in rectal fluids and tissues and low systemic exposure may be indicative of drug absorption mechanisms such as efflux transporters or metabolizing enzymes present in the rectal mucosa. Unlike the intracellular modification required for TFV and TAF to convert to its active form (TFV-DP), EVG is immediately bioavailable and active when present in fluids and tissues. The rapid detection of EVG in rectal fluids within 2 h at levels several orders of magnitude higher than in vitro IC_50_ (0.5–2.2 ng/mL) [37] suggests the on-demand applicability of the developed suppositories. The concomitant and rapid appearance of EVG in rectal fluids and tissue further support the utility and development of EVG-containing suppositories for on-demand rectal PrEP/PEP.

Encouraged by the PK profile of the PEG-based TFV/EVG (40/40 mg) suppositories, we evaluated the utility of combining TAF and EVG as a lower dose rectal suppository option for HIV prevention. Despite a five-fold difference in dosing (8 vs. 40 mg), no significant reduction in EVG exposures in the rectal fluid and tissue was observed, suggesting EVG may have reached saturation at the site of dosing. While TFV-DP concentrations in rectal tissues at 5 h were similar to those in macaques dosed with TFV/EVG (40/40 mg), TFV-DP levels were lower at 24 h suggesting rapid conversion of TAF to TFV may have resulted in decreased tissue loading capacity. Importantly, detection of TFV-DP in rectal lymphocytes isolated from distal tissues is noteworthy as drug distribution beyond the application point will be critical for rectal protection. Overall, our findings support further preclinical assessment of rectal efficacy by TFV/EVG and TAF/EVG suppositories in a validated macaque challenge model. These studies will be important to define protective efficacy and windows of protection for pre- and post-exposure dosing, all critical for informing dose selection and dosing modality in clinical trials.

User desirability of new dosage forms for HIV prevention is important. Vaginal and rectal inserts, as well as enemas containing ARVs for on-demand use, have been developed and show promising preclinical data in non-human primates [28,30,31,38]. A recent clinical trial was completed to better understand the likelihood of future product use and adherence to rectal administration of different dosage forms. The acceptance, tolerability, and adherence of three placebo dosage forms (insert, douche, and suppository) were evaluated by participants that engage in RAI practices (MTN035; ClinicalTrials.gov Identifier: NCT03671239). Data showed options are important when it comes to products for preventing HIV from anal sex. Efficacy was the strongest determinant of product choice (30%), followed by delivery method (18%) and side effects (17%) [39]. Other factors such as the timing of use around sex, duration of protection, frequency of use, and the need for a prescription were also considered. While an efficacious douche (>95% efficacy) that could be used within 30 min before sex and offers 3–5 days of protection was highly desired, a fast-dissolving rectal suppository that is discreet and has potential as an alternative to sexual lubricant was considered the best option for some. Overall, the ranking of the most preferred rectal products was based on personal experiences and tradeoffs users might make in real-life situations [39].

## 5. Conclusions

In this work, we demonstrated the utility of novel rectal suppositories co-formulated with potent antiretroviral drugs as a promising drug delivery platform for rectal microbicides. The achieved in vivo mucosal levels of TAF, EVG, TFV, and TFV-DP support advancing the suppository dosage form for on-demand pre- and post-exposure prophylaxis.

## Figures and Tables

**Table 1:** Characterization of placebo suppositories.

Suppository Bases	Weight (g) (avg ± SD) *n* = 3	Hardness (kgs) (avg ± SD) *n* = 4	Disintegration (min) Time Ranges *	Thermal Transition Using DSC (°C) *n* = 2
Suppocire A	1.18 ± 0.01	2.83 ± 0.28	7–8	37.79
Witepsol H15	1.22 ± 0.01	5.08 ± 0.98	6–8	34.77
Cocoa Butter	1.18 ± 0.01	0.38 ± 0.29	3–4	32.97
PEG 8000/400 (60:40)	1.48 ± 0.01	2.57 ±0.21	12–15	56.39
PEG 3350/1000 (25:75)	1.50 ± 0.01	2.05 ± 0.20	8–9	37.86
PEG 3350/1000/400 (60:30:10)	1.49 ± 0.01	1.31 ± 0.14	7–8	55.08

* Disintegration time was noted from initiation to end of melting by visually observing changes in melting.

**Table 2:** Pharmacokinetic summary in macaques following rectal administration of TFV/EVG (40 mg/40 mg) suppositories formulated in witepsol H15 or PEG base.

	Tenofovir			Elvitegravir		
PK Parameter	C_max_, Median (range)	T_max_	AUC_0–24_	C_max_, Median (range)	T_max_	AUC_0–24_
Plasma	(ng/mL)	(h)	(ng × h/mL)	(ng/mL)	(h)	(ng × h/mL)
PEG	24.5 (14–106.0)	0.5	29.7	BLOQ (BLOQ–15)	-	5.6
Witepsol	BLOQ (BLOQ–22.0)	0.5	27.8	BLOQ (BLOQ–8.0)	-	5.1
Rectal Fluid	(µg/mL)	(h)	(µg × h/mL)	(µg/mL)	(h)	(µg × h/mL)
PEG	13.4 (0.63–71.2)	2	26.4	17.3 (1.6–37.0)	2	15
Witepsol	16.3 (14.4–24.4)	2	12.2	24.6 (15.1–27.5)	2	15.6
Biopsy	(ng/mg)	(h)	(ng × h/mg)	(ng/mg)	(h)	(ng × h/mg)
PEG	109.1 (BLOQ–945.6)	2	124	45.5 (BLOQ–253.1)	2	42.3
Witepsol	42.6 (26.5–253.1)	2	107.1	15.6 (9.2–184.4)	2	58.7

LLOQ in plasma (TFV and EVG) = 10 and 5 ng/mL, respectively. LLOQ in rectal fluid (TFV, EVG) = 10 ng/mL. LLOQ in rectal tissue (TFV, EVG) = 1 ng/mg.

**Table 3:** Pharmacokinetic summary in macaques following rectal administration of PEG-based suppositories containing TAF/EVG (8 mg/8 mg).

	Rectal Fluid			Tissue (Biopsy)		
Variable	C_max_, µg/mL, Median (Range)	T_max_, h	AUC_0–24_, µg × h/mL	C_max_, ng/mg, Median [Range]	T_max_, h	AUC_0–24_, ng × h/mL
TAF (8 mg)						
PEG suppository	0.019 (BLOQ–0.210)	2	0.03	BLOQ (BLOQ–4.0)	2	0.67
TFV (from TAF)						
PEG suppository	12.2 (0.284–48.1)	2	12.1	39.5 (BLOQ–82.0)	2	13.9
EVG (8 mg)						
PEG suppository	9.99 (0.457–22.4)	2	7.6	53.0 (BLOQ–127.0)	2	36.3

LLOQ in rectal fluid (TFV, EVG, TAF) = 10 ng/mL. LLOQ in rectal tissue (TFV, EVG, TAF) = 1 ng/mg.
